# Supplementary material for: Characterization of the Mechanism of Action of Serratia rubidaea Mar61-01 against Botrytis cinerea in Strawberries
Source: Plants (Basel). 2022 Dec 29;12(1):154. doi: 10.3390/plants12010154 (PMC9823761; doi:10.3390/plants12010154)
Supplement: Supplementary file 1 [file plants-12-00154-s001.zip › plants-2075738-supplementary.pdf]

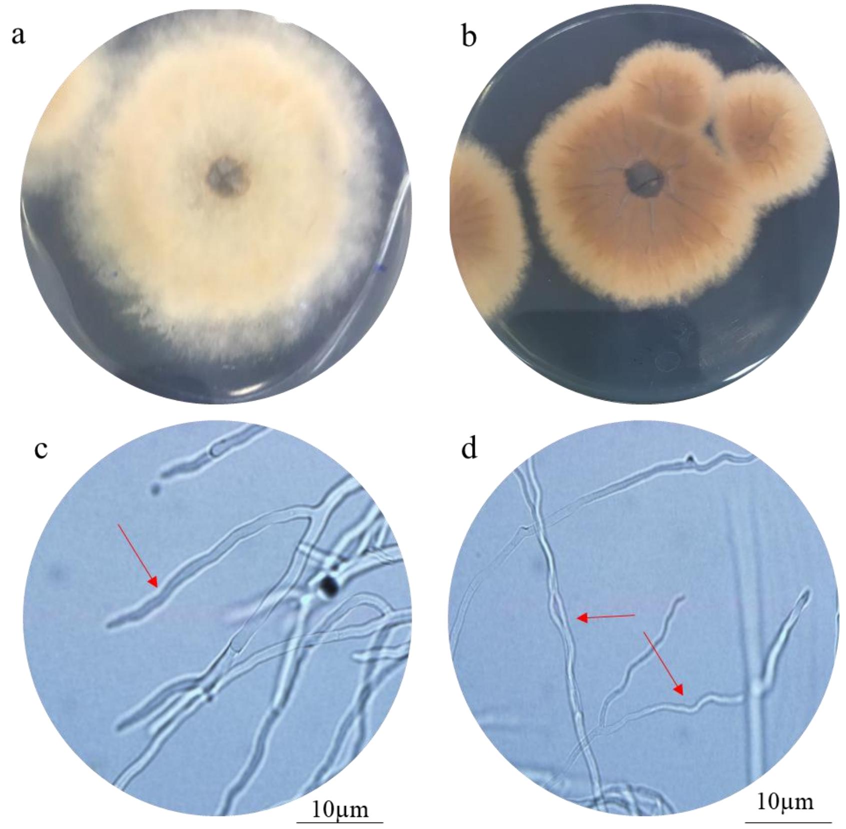

Figure S1. Effects of volatile organic compounds of *Serratia rubidaea* Mar61-01 on *Botrytis cinerea* mycelial growth. a) Control, b, c and d) treatment with *Serratia rubidaea* Mar61-01. Arrows indicate twisting in the hyphae and B) indicate shrinkage in the colony of fungal pathogen.

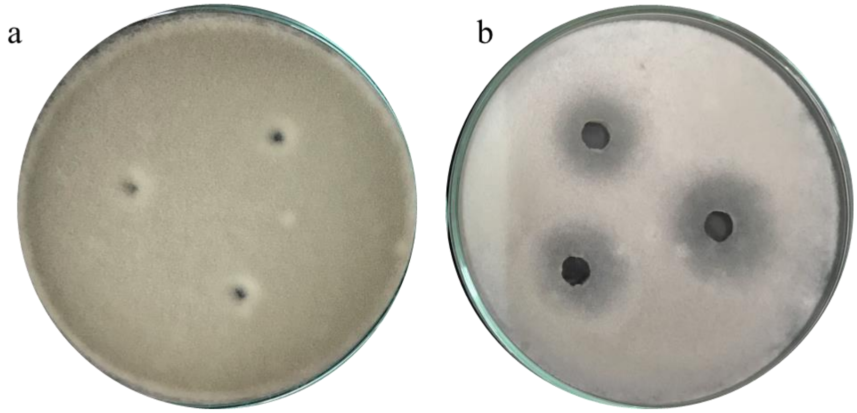

Figure S2. Effects of prodigiosin on mycelial growth of fungal pathogen a) Control and b) inhibited growth around the holes in treatment with prodigiosin

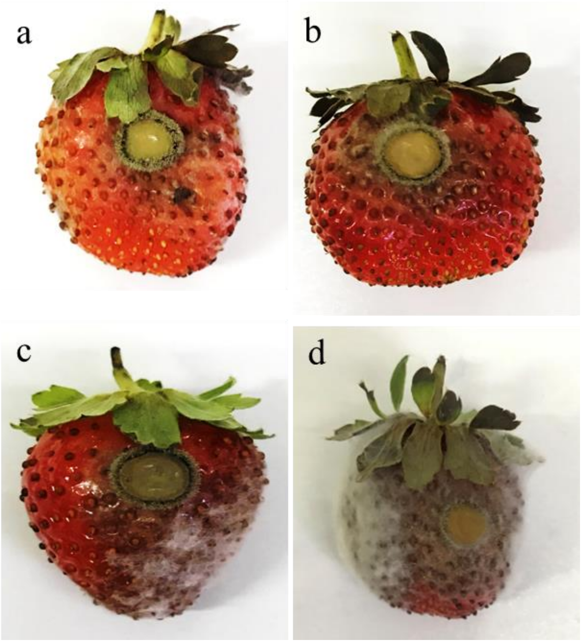

Figure S3. Disease severity on a and b) +prodigiosin+*B. cinerea* (BP) treatment and c and d) mock-treated control, 72 and 96 h after inoculation respectively.
